# Associations between Dietary Patterns and Physical Activity with Physical Fitness among Adolescents in Shandong Province, China: A Cross-Sectional Study

**DOI:** 10.3390/nu15061425

**Published:** 2023-03-15

**Authors:** Sizhu Wu, Xiaolei Xiu, Qing Qian

**Affiliations:** Department of Medical Data Sharing, Institute of Medical Information/Library, Chinese Academy of Medical Sciences & Peking Union Medical College, 3 Yabao Road, Chaoyang District, Beijing 100020, China

**Keywords:** dietary patterns, physical activity, physical fitness, parents’ educational level, adolescents, China

## Abstract

Background: The trend of physical fitness (PF) and physical activity (PA) among Chinese adolescents is not optimistic, and unhealthy dietary behaviors are common. PA and dietary patterns (DPs) have been linked to PF in adolescents, but the associations between DPs and PF with PF in Chinese adolescents are rarely discussed. Methods: A total of 8796 adolescents aged 11–18 were enrolled from Shandong Province, China. The CNSPFS battery was applied to assess PF. PA levels and diet quality were determined using the Physical Activity Questionnaire for Adolescents and the modified Chinese Diet Quality Questionnaire, respectively. This study used factor analysis to identify DPs and linear regression models to investigate the association between PF and related factors. Results: The average PF score of the participants was 75.67. Adolescents who were girls, lived in rural areas and were active in PA performed better on the PF test (*p* < 0.05). Boys whose fathers were university educated or above had a higher probability of achieving higher PF scores (OR 4.36, 95% CI 1.32–14.36); however, if their mothers were university educated or above, they had a lower probability of achieving higher PF scores (OR 0.22, 95% CI 0.063–0.76). Unhealthy dietary pattern was negatively correlated with cardiorespiratory fitness in boys (OR 0.56, 95% CI 0.31–0.98). The association between unhealthy dietary pattern and girls’ BMI became significant after adjustment for PA (*p* < 0.05). Conclusions: Girls performed better in PF than boys. Highly educated fathers could contribute to improve the PF performance in boys. There were four DPs among adolescents in Shandong Province, and different DPs may have different effects on PF in boys and girls.

## 1. Introduction

Physical fitness (PF) is a set of health- or skill-related attributes [1]. The components of health-related PF include body composition, muscular endurance, muscular strength, cardiovascular endurance and flexibility, which are widely recognized as markers of health-related outcomes throughout life [2,3]. The adolescence period is a crucial time for PF development. Improving PF in adolescents is of great significance not only to improving their academic performance, quality of life, cognitive ability and mental health but also to improving the quality of national health [4,5,6]. However, the decline of PF in adolescents has become a global public health problem. In recent years, although the PF of Chinese adolescents has generally improved, the trend of PF is still not optimistic. According to the results of the 8th National Survey on Student Physical Fitness and Health released by the Chinese Ministry of Education in 2021, only 17.7% of students aged 13–22 could achieve a “good” or “excellent” rating of PF [7]. Therefore, given the current situation of PF among Chinese adolescents, it is necessary to take effective measures to improve their PF.

There are various factors that affect adolescents’ PF, such as genetics, biological characteristics, dietary habits and physical activity (PA) [8,9,10]. Studies have shown that PA and diet are associated with PF in adolescents. Relevant research can be divided into the following three categories. (1) Association of PA and diet with weight status: The results of several studies have indicated that intermittent fasting is beneficial for body weight, but diet-induced weight changes are generally short-lived and greater benefits can be gained through vigorous PA [11,12]. Bogataj et al. have demonstrated that just 8 weeks of school-based high-intensity interval training with three sessions a week and nutrition intervention can improve upper body muscle and physical aerobic performance in adolescents and help reduce BMI in overweight girls [13]. Oh et al. identified dietary patterns in Korean children and adolescents, and found that the “fast food and soda pattern” was positively associated with waist circumference, serum insulin and BMI, while the “white rice and kimchi pattern” and “oil and seasoned vegetables pattern” indicated a preventive effect on these parameters [14]. (2) Effect of diet and PA on health: Previous studies have found an effect of diet on cardiorespiratory fitness (CRF) and metabolic syndrome (MetS) [15,16]. For example, CRF was positively associated with frequent consumption of fruits, vegetables, bread and dairy products [15,17]. Shahinfar et al. showed that adherence to the mixed dietary pattern was associated with increasing odds of MetS in Iranian adults [18]. Moreover, Wenjie et al. found that adequate calcium intake and the improved CRF were essential for the development of good mental health in adolescents aged 12–13 years [19]. (3) Relationship between Mediterranean diet and PA and different parameters of PF: The Mediterranean diet (MD) is postulated as one of the healthiest dietary patterns that exists [20]. The authors concluded that optimal adherence to the MD pattern was associated with higher CRF and PA levels as well as high levels of muscular strength [21,22,23,24]. For an instance, Cristina et al. found that greater adherence to the MD pattern was related with higher CRF and lower limbs muscular strength and speed agility [25]. Pablo et al. also showed that higher levels of CRF in boys and girls were associated with medium and high adherence to the MD [26]. However, some studies indicated the positive correlation between the MD pattern and higher CRF and speed agility only in boys [27,28].

Although several studies have analyzed the associations between dietary patterns and PA with PF among adolescents, there are still several problems. First, the existing studies are controversial about the correlation between parents’ educational level and PF. One study showed that the educational level of German parents was positively correlated with their children’s aerobic fitness [29]. However, a Swiss study found that parents’ educational level affected children differently, with the mother’s education level appearing to have a greater impact on children’s PF [30]. Another study found that those Catalan children whose fathers had higher education had lower waist circumference and BMI [31]. Second, the measurement standards of Chinese and foreign students’ PF are different. For an instance, Spain measures upper body muscular strength of students using a hand dynamometer with adjustable grip, whereas China does not measure girls’ upper body muscle strength and only uses pull-ups to measure the upper body muscular strength of boys [24,32]. Third, most studies on the relationship between dietary patterns and PF have focused on the MD pattern. The MD pattern is characterized by high consumption of fruits and vegetables, nuts, cereals, fish and olive oil and minimal amounts of red meat and dairy products, which is quite different from the grain-based Chinese dietary pattern [25]. Currently, we have not found any reports on the interaction between dietary patterns and PF in Chinese adolescents. Therefore, it is necessary to explore the relationship between dietary patterns, PA and PF among Chinese adolescents based on Chinese physical fitness measurement standards and dietary culture.

This study was designed with the following general aims: (1) to comprehensively evaluate the physical fitness status of adolescents (11–18 years old) in Shandong Province; (2) to explore the relationship between diet, physical activity and physical fitness; and (3) to further analyze the differences between boys and girls in the relationships between dietary patterns, physical activity and different parameters of PF. We hope that the findings of this study can provide a valid and specific theoretical basis for improving the physical fitness of Chinese adolescents.

## 2. Materials and Methods

### 2.1. Participants and Study Design

This is a cross-sectional study that is part of a special program to investigate the status quo of health and health-related behaviors of Chinese junior and senior high school students. Detailed sampling methods have been published elsewhere [33]. Briefly, the sample for this study was selected using a probability-proportional-to-size sampling method during the 2020–2021 semester. Ultimately, 11,063 adolescents aged 11–18 years were recruited for the study. After excluding illogical samples and those missing information on PF or diet, a total of 8796 participants were finally included for analysis. Teachers, parents and students filled out a consent form prior to enrollment in this survey. The research was approved by the Ethics Committee of Shandong University, China (20180517).

### 2.2. Data Availability

The data used in this study are publicly available in the Population Health Data Archive [34].

### 2.3. Measurements

#### 2.3.1. Demographic Factors

They included gender, age, place of residence (rural/urban), parents’ educational level (junior high school and below, high school, university and above), and family economic status (poor, middle, good). This information was collected through a self-reported questionnaire for participants.

#### 2.3.2. Physical Activity Measure

PA was evaluated by the Physical Activity Questionnaire for Adolescents (PAQ-A). This scale is a revised version of the Physical Activity Questionnaire for Children (PAQ-C), which is designed to assess the PA level of adolescents [35]. It is on a 5-point scale (1–5), with higher scores indicating higher levels of PA. The results can be divided into two categories: low PA levels (1–1.9 points) and high PA levels (2–5 points) [36]. The validity and reliability of the PAQ-A has been validated among Chinese adolescents [37]. This study also demonstrated that the questionnaire has good internal consistency and structural validity: the Cronbach’s alpha value was 0.82; the Kaiser—Meyer—Olkin (KMO) value was 0.83; and for Bartlett’s test, *p* < 0.001.

#### 2.3.3. Dietary Assessment

Participants’ dietary status was assessed using the modified Chinese Diet Quality Questionnaire (DQQ), a 5-point scale (1–5) for rapid qualitative and quantitative analysis of participants’ diet quality. The DQQ contains three diet scores: GDR-Healthy score, GDR-Limit score and overall GDR score. The GDR-Healthy score reflects global recommendations for health-protective foods in healthy diets. The GDR-Limit score reflects global recommendations for limiting dietary components. Lower overall GDR score, lower GDR-Healthy score and higher GDR-Limit score indicate poorer diet quality [38]. Studies have shown that the DQQ can be a valid tool for assessing the diet quality of Chinese children and adolescents aged 7–18 [39]. The DQQ includes 29 food groups. Considering the dietary habits of people in Shandong Province and the purpose of this study, we removed cereals and cheese from the DQQ. Additionally, the food items were combined into 17 groups in this study to facilitate adolescents filling out the questionnaire. Each participant was required to answer the question (How often have you eaten this type of food in the past week?), and the answers were divided into five levels (1 = “0 times”, 2 = “1–2 times”, 3 = “about once every 2 days”, 4 = “about once a day”, or 5 = “more than once a day”). In this study, the Cronbach’s alpha was 0.87, and the questionnaire was demonstrated to have good construct validity (KMO 0.91, Bartlett’s test *p* < 0.001).

#### 2.3.4. Physical Fitness

Trained physical education teachers administered all PF tests according to standard operating procedures The training was conducted through workshops and the specific procedures for data collection have been described in a previous paper [33]. PF was assessed by the Chinese National Student Physical Fitness Standard (CNSPFS) battery published by the Ministry of Education of the People’s Republic of China, which is a reliable and valid instrument for assessing PF in adolescents [40]. The total score of the participants’ PF test is 100 points, and the percentage of the score for each item and the test method are shown in Table 1.

### 2.4. Statistical Analysis

All data were analyzed by IBM SPSS Statistics Version 26.0 (IBM Corporation, Armonk, NY, USA). Continuous variables were expressed as mean (M) and standard deviation (SD), and numbers (N) and percentages (%) were reported for categorical variables. Independent samples 2-tailed *t*-test, one-way analysis of variance (ANOVA) test or Chi-square test were used to compare the differences between groups of categorical variables, as appropriate. Furthermore, we performed multiple comparisons using Bonferroni-corrected *p*-values to account for inflation of type-I errors due to multiple comparisons made. To investigate the relationship between predictors (diet and PA) and outcomes (PF), we first analyzed their relationship using linear regression. Then, factor analysis was used to analyze the dietary patterns of the participants. As an exploratory analysis, we further investigated the differences in the relationship between PF and dietary patterns in boys and girls. Specifically, each PF was regressed on the dietary patterns after adjusting for basic confounders such as age, place of residence, parents’ educational level and family economic status (Model 1). Other analyses adjusted for high PA levels (Model 2). Odds ratios and their 95% confidence intervals obtained from the model were reported. A *p*-value <0.05 was considered to be statistically significant.

## 3. Results

### 3.1. Sociodemographic Characteristics of the Participants

In total, 8796 students (4332 (49.2%) boys; 4464 (50.8%) girls) were included in the final statistical analysis of this study. The demographic characteristics of the participants are summarized in Table 2. The mean age of the participants was 14.32 years and mean PF score was 75.67. The results showed significant differences in PA, diet, and PF between boys and girls. A significantly higher percentage of boys than girls were active in PA (58.1% vs. 46.5%). However, the proportion of boys with a BMI above normal was significantly higher than that of girls (27.4% vs. 21.3%). Girls had a significantly higher mean overall GDR score (7.53 vs. 7.45) and mean PF score than boys (77.34 vs. 73.94).

**Figure 1:**
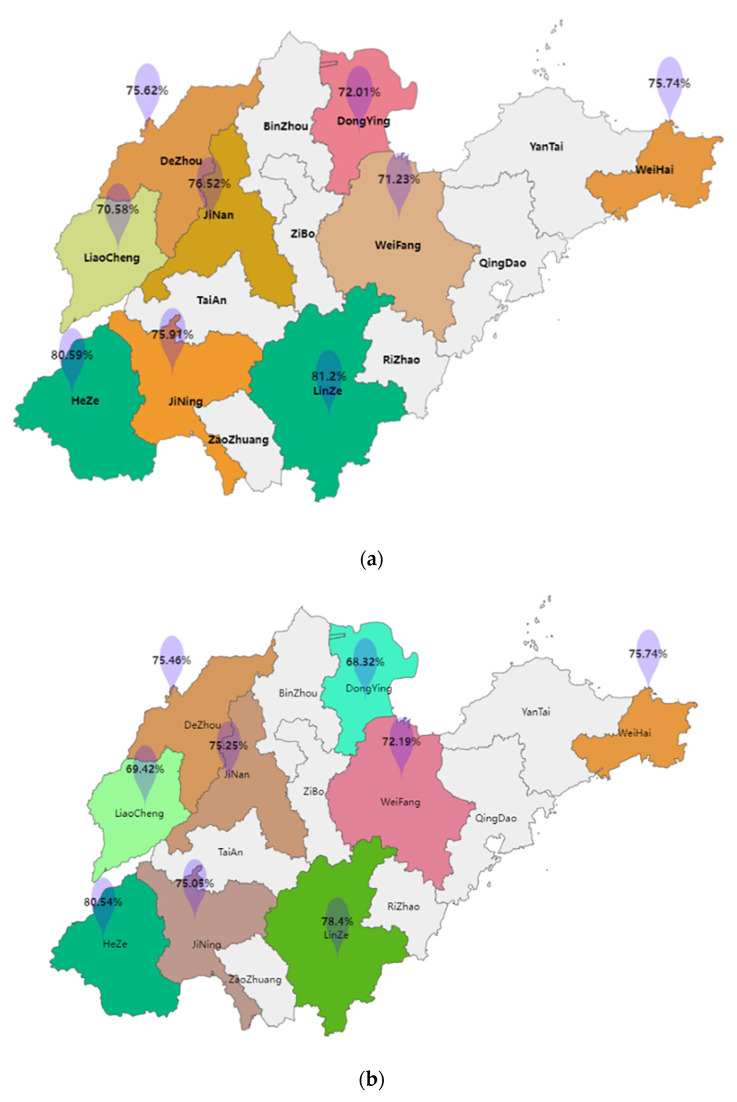

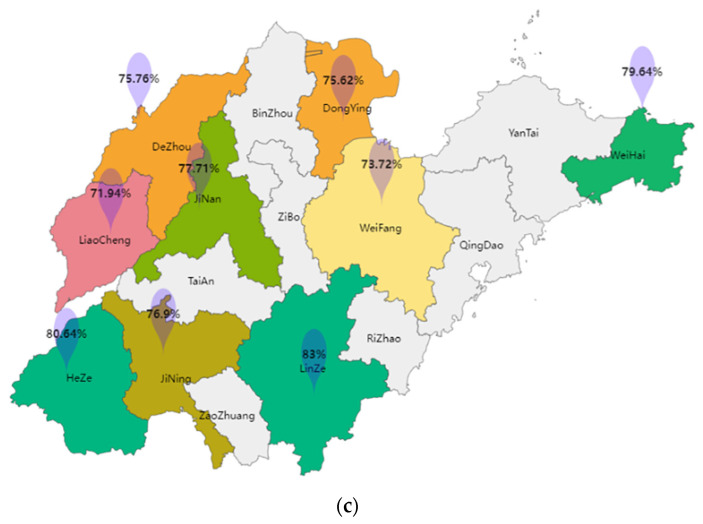
Average scores of physical fitness tests for boys and girls by city level in Shandong Province. (**a**) The average score of all participants in the physical fitness test. (**b**) The average score of the physical fitness test for boys. (**c**) The average score of the physical fitness test for girls.

The mean PF scores of adolescents varied widely among cities in Shandong province, ranging from 70.58 in Liaocheng to 81.2 in Linyi (Figure 1). In terms of boys’ PF, boys in Heze had the highest mean PF score of 80.54, while boys in Dongying had the lowest mean PF score of 68.32. Girls in Linyi had the highest mean PF score of 83.

### 3.2. Physical Fitness Status of Adolescents in Shandong Province

**Figure 2:**
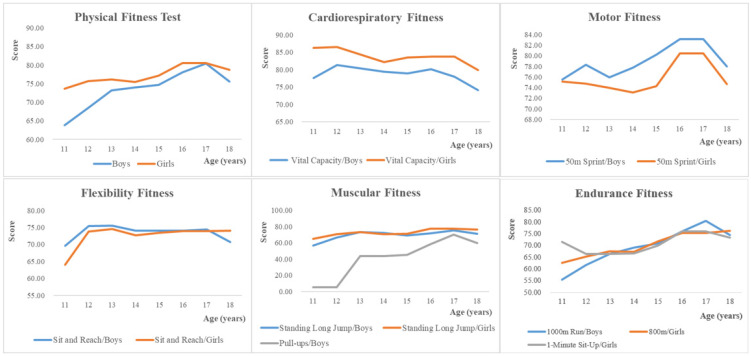
Age-related differences in different physical fitness tests expressed as standardized scores.

Table 3 shows the means and deviations of the PF tests by age and gender, and the variation of boys’ and girls’ performance on the various PF tests with age is shown in Figure 2. It can be observed that girls had higher PF scores than boys in all age groups. In particular, girls performed significantly better than boys on the CRF test. Boys performed better than girls on the motor test. However, boys had poorer upper body muscular strength and only the 17-year-old boys passed the pull-ups test with an average score (70.07).

### 3.3. Relationship between Physical Fitness and Other Variables

In this study, the PF score was used as the dependent variable, and the relationship between PF and various factors was analyzed by multiple linear regression (Table 4). The results showed that girls’ PF performance was 26.38 times (OR 26.38, 95% CI 16.97–41.01) higher than that of boys. Adolescents living in rural areas had significantly higher PF scores than those living in urban areas, and this phenomenon was more pronounced among boys (OR 8.00, 95% CI 3.89–16.45). Furthermore, we found that participants’ PF scores became higher as participants aged. The PF scores of boys whose fathers had a university or above level of education were 4.36 times (OR 4.36, 95% CI 1.32–14.36) higher than those whose fathers had a junior high school or below level of education. Compared to boys whose mothers had a junior high school or below level of education, boys whose mothers had a university or above level of education had 78% lower (OR 0.22, 95% CI 0.063–0.76) PF scores. A statistically significant positive correlation was found between PF scores and PA levels (*p* < 0.01).

### 3.4. Dietary Patterns of Adolescents Aged 11–18

**Figure 3:**
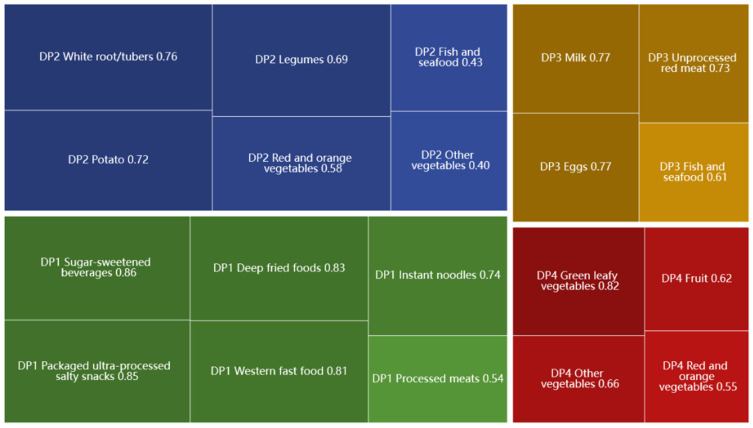
Factor loadings of various food items in each dietary pattern.

**Figure 4:**
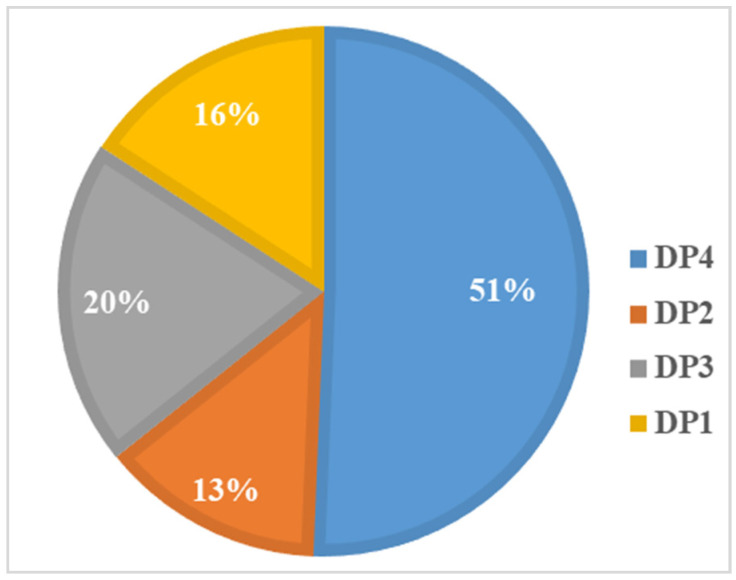
Percentage of each dietary pattern.

**Figure 5:**
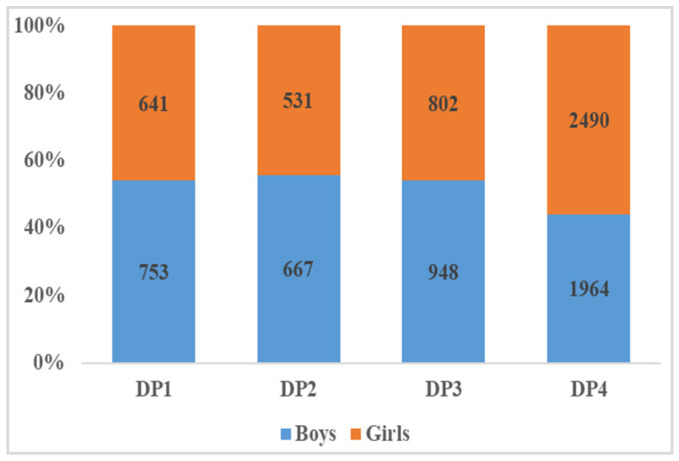
Number and proportion of boys and girls in each dietary pattern.

The study conducted factor analysis (based on principal component analysis) to investigate participants’ dietary patterns (DPs) based on the percentage energy intake of 17 food groups. To determine the number of DPs, we considered eigenvalues greater than 0.4 and scree plots. Finally, four mutually exclusive DPs were identified, as shown in Figure 3. The DP was named according to salient food characteristics. DP1 was named the “unhealthy food pattern”, and was characterized by high factor loading from sugar-sweetened beverages, packaged ultra-processed salty snacks, deep-fried foods, Western fast-food, instant noodles and processed meats. Similarly, the other three DPs were named DP2—tuber and legume pattern (white root/tubers, potato, legumes, red and orange vegetables), DP3—protein and seafood pattern (eggs, milk, unprocessed red meat, fish and seafood), DP4—vegetable and fruit pattern (green leafy vegetables, other vegetables and fruit). These four DPs could explain 22.67%, 15.29%, 13.89% and 12.47% of the variance, respectively. Food variables with higher factor loadings in the DP indicated higher intake. Each participant had four DP scores, with the largest DP score indicating their preference for that DP. As shown in Figure 4 and Figure 5, 50.6% of the participants tended to be DP4, and 55.90% of them were girls; 15.8% of participants preferred DP1, of which 54.1% were boys.

### 3.5. Associations between Dietary Patterns and Physical Fitness

Table 5 showed the results of the linear regression analysis of the predictor (DPs) for different parameters of PF after adjusting for the basic confounders (age, place of residence, parents’ educational level and family economic status; model 1) and additional adjustment for PA confounders (model 2). DP1 was negatively correlated with CRF in boys (OR 0.56, 95% CI 0.31–0.98, *p* < 0.05). DP2 was negatively correlated with BMI in girls (OR 0.84, 95% CI 0.72–0.97, *p* < 0.05) and positively correlated with flexibility fitness in boys (OR 3.03, 95% CI 1.36–6.77, *p* < 0.01). DP3 was positively associated with girls’ motor fitness (OR 2.37, 95% CI 1.14–4.95, *p* < 0.05) and boys’ upper body muscular strength (OR 1.15, 95% CI 1.04–1.27, *p* < 0.01). When analyzed with additional adjustment for PA (model 2), all results remained statistically significant (all *p* < 0.05). Moreover, the relationship between DP1 and girls’ BMI became significant (OR 1.12, 95% CI 1.001–1.26, *p* < 0.05) after adjusting for age, place of residence, parents’ educational level, family economic status and PA.

## 4. Discussion

The purpose of this study was to examine the associations between PA levels, DP and PF among Chinese adolescents, taking into account demographic factors such as gender, age, place of residence, parents’ educational level and family economic status. We had several important findings.

### 4.1. Physical Fitness

The average PF score of the participants was 75.67, and 39.4% of participants could achieve a “good” or “excellent” rating from PF. Compared with 2016, the PF performance of adolescents in Shandong Province has been greatly improved. Specifically, their muscular endurance and muscular strength improved. For example, the 1000 m/800 m run was shortened by 8.1 s and 5.5 s for boys and girls, respectively, and the standing long jump was improved by 7.51 cm and 3.00 cm, respectively. Additionally, the average number of pull-ups for boys also increased by 1.18 [41]. However, 55.8% of the boys still failed the pull-ups test, and even 31% scored 0. The boy’s upper body muscular strength is worrying and it is necessary to carry out targeted exercises for boy’s upper body muscles. Secondly, we found statistical differences in PF by gender, place of residence and PA. We observed that girls had better PF performance than boys, especially in CRF. Adolescents living in rural areas had higher PF scores than those living in urban areas; active PA could significantly improve adolescents’ PF performance, and PF performance improved with age. These findings are consistent with previous studies [42,43,44]. However, the PF scores of 18-year-old boys in this study were significantly lower than those of 17-year-old boys. This result may be due to the small sample size of 357 (4.1%) for 18-year-old boys. The specific reasons for this will be explored in detail in the future by further expanding the sample size.

It is noteworthy that parents’ educational level was only associated with boys’ PF in this study. Boys whose fathers had a university or above level of education performed significantly better in PF than those whose fathers had a junior high school or below level of education. However, boys whose mothers had a university or above level of education performed worse in PF than those whose mothers had a junior high school or below level of education. These findings are inconsistent with the findings of previous studies [29,30]. These discrepancies may be partly attributable to the different division of parenthood in Chinese families. Chinese fathers play a key role in their children’s PA choices and behaviors [45]. Adolescents whose fathers were highly educated engaged in significantly more PA per week than those whose fathers were less educated, and PA levels were positively associated with adolescents’ PF performance [46,47]. However, Chinese mothers, especially those from better-off and better educated families, invest more in their children’s education and are more willing to enroll their children in cram schools, which greatly reduces their children’s PA time [48].

### 4.2. Dietary Patterns

Four DPs were identified in this study, including the unhealthy food pattern, tuber and legume pattern, protein and seafood pattern and vegetable and fruit pattern. It can be found that the DPs derived from this study are significantly different from the Korean DP and MD patterns [14,25]. According to the recommendations of the “Dietary Guidelines for Chinese School-aged Children (2022)”, school-age children should drink milk every day and not drink sugary drinks. However, we found that 15.8% of the participants preferred the “unhealthy food pattern”, and only about half of them (58.8%) drank milk on a daily basis. It is evident that the milk consumption among Chinese adolescents is relatively low, and the promotion of scientific consumption concepts of dairy products should be strengthened to raise the correct awareness of dairy products among adolescents and their parents.

Additionally, 24.3% of participants in this study were overweight, which is higher than the findings in 2017 [49]. Studies have shown that the prevalence of overweight among Chinese adolescents continues to increase [50]. Furthermore, we found that GDR scores were associated with BMI in Chinese adolescents, with higher GDR-limit scores associated with higher odds of obesity and higher overall GDR scores associated with lower odds of obesity [38]. However, the overweight participants in this study consumed more unhealthy foods than those who were obese. This may be due to misperceptions about their weight among overweight individuals. Some studies have reported that adolescents who perceive themselves to be heavier have more restrictions on their diets [51]. Therefore, in order to effectively curb the epidemic of overweight and obesity among Chinese adolescents and to promote their healthy growth, it is necessary to enhance the dissemination of nutritional health knowledge among Chinese adolescents, especially boys, and guide them to develop the correct view of health.

### 4.3. Physical Activity, Dietary Patterns and Physical Fitness

Further analysis of different PF tests revealed that DPs had different effects on the PF of boys and girls. Energy-dense and low-nutrient junk food is a major component of DP1. Excessive consumption of junk food can lead to excessive accumulation of energy, obesity and other metabolic diseases [43]. Another investigation reported that excessive consumption of junk food in obese school children is associated with derangement of sympathetic cardiovascular functions and reduced pulmonary functions [52]. Our findings have showed that DP1 was negatively correlated with CRF in boys. This may be due to the higher prevalence of obesity among boys in this sample. Whether there are gender differences in the effect of junk food on CRF among Chinese adolescents needs to be further explored. DP2 (tuber and legume pattern) is rich in white root/tubers, potato, legumes, red and orange vegetables and low in seafood. It is a major food source of isoflavones, phytosterols, lecithin, chlorophyll, lutein, lycopene, anthocyanins, polyunsaturated fatty acids and dietary fiber [53,54]. Previous studies have shown that there was a negative association between DP2 and abdominal obesity among adolescents [16]. However, in the present study, DP2 was only negatively associated with BMI in girls but not significantly associated with BMI in boys. In addition, moderate intake of high-quality protein has been shown to help improve muscular strength and speed-agility in adolescents [55]. However, we found that DP3 (protein and seafood pattern) had different effects on PF in boys and girls. For girls, DP3 was positively associated with motor fitness, while for boys, DP3 was positively correlated with upper body muscular strength. To examine the role of PA on the relationship between DPs and PF, we further adjusted the analysis for active PA. The results showed that the relationship between DP1 and girls’ BMI became significant after adjustment for active PA. One explanation is that physically active individuals make healthier and more beneficial food choices in order to perform better [56]. However, we did not find a significant change in the relationship between DP1 and boys’ BMI due to adjustment for active PA. In the future, more investigations are needed to explore this cause.

### 4.4. Study Limitations and Future Research

Some limitations in this study must be explained. First, this study was based on participants’ self-reports, which may be subject to self-report bias. Second, although gender, age, place of residence, parents’ educational level and family economic status were accounted for in our analyses, residual confounding factors such as sedentary behavior and pubertal development status cannot be excluded. Third, it must be noted that this research is a cross-sectional study, so it cannot reflect the development and changes of PF in adolescents. Given the above limitations, longitudinal studies with more diverse sample sources and more comprehensive designs are needed in the future to better understand the influencing factors of Chinese adolescents’ PF and formulate corresponding countermeasures.

## 5. Conclusions

This paper elucidates for the first time the impact of DPs and PA on the PF through the factor analysis of adolescents aged 11–18 years in Shandong Province from 2020–2021, and further analyzes the differences between boys and girls. We have found that: (1) Overweight, unhealthy food patterns and poor upper body muscular strength were more common in boys than in girls. (2) Highly educated fathers contributed to improve PF in boys, but highly educated mothers did not. (3) Four DPs were obtained, and different DPs may have different effects on PF in boys and girls. Specifically, the unhealthy food pattern was negatively associated with CRF in boys. After adjusting for active PA, the relationship between unhealthy food pattern and girls’ BMI became significant. The tuber and legume pattern was negatively associated with BMI in girls, yet positively associated with flexibility fitness in boys. The protein and seafood pattern was positively associated with girls’ motor fitness and boys’ upper body muscular strength. Therefore, in order to effectively improve PF in adolescents, strategy developers should take into account the differences in diet and PF between boys and girls as well as the different effects of DPs and PA on PF in boys and girls so as to maximize the effectiveness of interventions. Our findings could have benefits in formulate a theoretical basis for the development of PF in Chinese adolescents. Future larger prospective studies are needed to explore the relationship between DPs, PA and PF and other health-related parameters in the Chinese adolescents.

## Figures and Tables

**Table 1:** Methods and scoring criteria of the physical fitness test for Chinese adolescents and the proportion of scores for each item.

Category	Test Indicator	Description	Scoring Criteria	Weight
**Body Shape**	Body Mass Index (BMI)	BMI scores are calculated as weight in kilograms divided by squared height in meters (kg/m^2^). Participants’ height (cm) is measured to the nearest 0.1 cm barefoot, and weight (kg) is examined to the nearest 0.1 kg by GMCS-IV (Jianmin, Beijing, China).	Normal = 100 Underweight = 80 Overweight = 80 Obese = 60	15%
**Cardiorespiratory Fitness (CRF)**	Vital Capacity (VC)	VC refers to the amount of air that the lungs can expel after having been filled completely and is measured with spirometry.	Excellent: 90–100 Good: 80–85 Pass: 60–78 Fail: ≤50	15%
**Motor Fitness**	50 m Sprint	The test requires students to start from a uniform starting point and records the time it takes for the student to run 50 m. Performance is recorded to the nearest 0.1 s.	Excellent: 90–100 Good: 80–85 Pass: 60–78 Failed: ≤50	20%
**Flexibility Fitness**	Sit and Reach (SR)	SR reflects flexibility level of the lower body. Each participant performs the SR test twice, and the score for the farthest distance (measured to the nearest 0.1 cm) is recorded.	Excellent: 90–100 Good: 80–85 Pass: 60–78 Fail: ≤50	10%
**Muscular Fitness**	Standing Long Jump	To test the explosive power and physical coordination of the lower limbs, subjects are allowed to make three attempts. The longest distance (in cm) is recorded as the official score.	Excellent: 90–100 Good: 80–85 Pass: 60–78 Failed: ≤50	10%
	Pull-ups/Boys	It indicates the upper body muscular endurance of the boys. The final score is recorded as the number of successful repetitions completed.	Excellent: 90–100 Good: 80–85 Pass: 60–78 Fail: ≤50	10%
**Endurance Fitness**	1-Minute Sit-Up (SU)/Girls	This is performed to assess the abdominal muscular endurance of the girls. The test instructs participants to perform sit-ups as many times as possible for one minute. The final score is recorded as the number of successful repetitions completed.	Excellent: 90–100 Good: 80–85 Pass: 60–78 Fail: ≤50	10%
	1000 m/800 m Run	To test the participants’ endurance fitness, boys and girls are instructed to perform a 1000 m run and an 800 m run, respectively. Running performance is recorded to the nearest 0.1 s.	Excellent: 90–100 Good: 80–85 Pass: 60–78 Fail: ≤50	20%

**Table 2:** Demographic characteristics of participants and differences between diet, PA and PF by participants’ gender, N = 8796.

Characteristics	N (%) or Mean ± SD			χ^2^/t	*p*
	All	Boys	Girls		
**Gender**					
Boys	4332 (49.2)				
Girls	4464 (50.8)				
**Age**	14.32 ± 1.82	14.22 ± 1.80	14.41 ± 1.84	−4.83	<0.001
**Place of residence**				1.24	0.265
Urban	4351 (49.5)	2169 (50.1)	2182 (48.9)		
Rural	4445 (50.5)	2163 (49.9)	2282 (51.1)		
**Father’s education level**				6.80	0.033
Junior high school and below	5563 (63.2)	2684 (62.0)	2879 (64.5)		
High school	1695 (19.3)	852 (19.7)	843 (18.9)		
University and above	1538 (17.5)	796 (18.4)	742 (16.6)		
**Mother’s education level**				2.35	0.309
Junior high school and below	5925 (67.4)	2890 (66.7)	3035 (68.0)		
High school	1492 (17.0)	738 (17.0)	754 (16.9)		
University and above	1379 (15.7)	704 (16.3)	675 (15.1)		
**Family economic status**				20.15	<0.001
Poor	1426 (16.2)	757 (17.5)	669 (15.0)		
Middle	6679 (75.9)	3200 (73.9)	3479 (77.9)		
Good	691 (7.9)	375 (8.7)	316 (7.1)		
**PA**				119.42	<0.001
Inactive	4205 (47.8)	1815 (41.9)	2390 (53.5)		
Active	4591 (52.2)	2517 (58.1)	2074 (46.5)		
Diet					
GDR-Limit score	16.42 ± 6.16	16.86 ± 6.53	16.00 ± 5.75	6.49	<0.001
GDR-Healthy score	23.91 ± 5.63	24.31 ± 5.79	23.53 ± 5.44	6.45	<0.001
Overall GDR score	7.49 ± 6.63	7.45 ± 6.82	7.53 ± 6.45	−0.56	<0.001
**Physical Fitness Score**	75.67 ± 10.94	73.94 ± 11.96	77.34 ± 9.56	−14.68	<0.001
**Height level classification**				116.53	<0.001
High	3826 (43.5)	1670 (38.6)	2156 (48.3)		
Middle	4691 (53.3)	2467 (56.9)	2224 (49.8)		
Short	279 (3.2)	195 (4.5)	84 (1.9)		
**BMI classification**				49.02	<0.001
Obese	951 (10.8)	553 (12.8)	398 (8.9)		
Overweight	1187 (13.5)	632 (14.6)	555 (12.4)		
Normal	6177(70.2)	2910 (67.2)	3267 (73.2)		
Under-weight	481 (5.5)	237 (5.5)	244 (5.5)		
**VC score**	81.54 ± 17.26	79.49 ± 17.52	83.53 ± 16.77	−11.05	0.131
**50 m Sprint score**	77.27 ± 18.07	78.88 ± 18.56	75.70 ± 17.43	8.26	<0.001
**SR score**	73.83 ± 17.70	74.31 ± 17.10	73.36 ± 18.26	2.51	0.015
**Standing Long Jump score**	72.04 ± 19.46	70.90 ± 20.36	73.15 ± 18.47	−5.41	<0.001
**Pull-ups score**		42.05 ± 35.76			
**1-Minute SU score**			69.99 ± 17.95		
**1000 m Run score**		69.43 ± 24.11			
**800 m Run score**			70.46 ± 20.89		

**Table 3:** The means and standard deviations of physical fitness tests by age and gender.

Age	Heigh (cm)	Body Mass Index	Vital Capacity (mL)	50 m Sprint (s)	Sit and Reach (cm)	Standing Long Jump (cm)	Pull-ups (n)	1-Minute Sit-Up (n)	1000 m Run (min)	800 m Run (min)
**Boys**										
11	156.66 ± 8.74	21.17 ± 4.59	2292.06 ± 724.77	9.31 ± 1.43	5.46 ± 7.13	164.30 ± 23.38	3.27 ± 6.91		5.13 ± 1.07	
12	161.10 ± 8.36	20.43 ± 3.98	2771.85 ± 829.97	8.90 ± 1.11	7.96 ± 9.55	178.55 ± 27.02	4.75 ± 7.04		4.95 ± 0.99	
13	166.30 ± 8.39	20.62 ± 4.21	3056.03 ± 881.19	8.67 ± 1.13	9.01 ± 9.12	187.71 ± 27.81	4.36 ± 5.53		4.78 ± 1.06	
14	169.20 ± 7.79	20.15 ± 3.86	3286.94 ± 785.87	8.29 ± 1.02	9.40 ± 7.91	201.60 ± 27.37	4.80 ± 5.45		4.45 ± 0.84	
15	172.04 ± 7.40	20.49 ± 3.74	3596.83 ± 891.85	7.96 ± 0.87	10.54 ± 7.07	211.04 ± 26.46	5.75 ± 5.37		4.22 ± 0.66	
16	175.44 ± 5.80	21.05 ± 3.58	3976.45 ± 1013.95	7.62 ± 0.69	11.80 ± 6.72	222.90 ± 23.92	9.19 ± 6.48		3.86 ± 0.57	
17	176.60 ± 5.25	21.19 ± 3.24	4083.37 ± 1066.03	7.53 ± 0.65	13.28 ± 6.36	232.28 ± 22.56	12.25 ± 6.33		3.63 ± 0.41	
18	176.51 ± 5.63	21.16 ± 3.52	4094.23 ± 1035.41	7.58 ± 0.60	12.41 ± 6.54	231.54 ± 21.42	11.20 ± 6.38		3.76 ± 0.53	
**Girls**										
11	156.72 ± 7.33	19.98 ± 3.99	2292.06 ± 724.77	9.42 ± 1.11	7.76 ± 8.13	155.02 ± 23.82		31.96 ± 10.66		4.35 ± 0.72
12	159.28 ± 6.66	19.75 ± 3.63	2771.85 ± 829.97	9.39 ± 1.22	11.24 ± 7.75	161.58 ± 21.82		29.46 ± 10.85		4.30 ± 0.71
13	161.74 ± 5.89	19.90 ± 3.59	3056.03 ± 881.19	9.36 ± 1.17	12.69 ± 9.18	164.54 ± 22.03		30.34 ± 10.10		4.25 ± 0.67
14	163.09 ± 5.73	19.90 ± 3.51	3286.94 ± 785.87	9.30 ± 1.09	12.78 ± 7.81	165.84 ± 22.38		31.44 ± 9.83		4.17 ± 0.79
15	163.17 ± 5.34	20.41 ± 3.13	3596.83 ± 891.85	9.17 ± 1.00	13.82 ± 7.49	168.29 ± 20.96		33.81 ± 9.68		3.97 ± 0.56
16	164.67 ± 5.12	20.12 ± 2.88	3976.45 ± 1013.95	8.67± 0.93	14.60 ± 6.74	176.63 ± 18.06		39.18 ± 10.40		3.78 ± 0.48
17	164.73 ± 4.91	20.07 ± 2.82	4083.37 ± 1066.03	8.62 ± 0.92	15.16 ± 6.45	178.73 ± 16.90		39.45 ± 9.71		3.66 ± 0.38
18	164.04 ± 4.71	20.45 ± 2.81	4094.23 ± 1035.41	8.85 ± 0.94	15.61 ± 5.85	176.75 ± 17.45		39.24 ± 10.29		3.69 ± 0.42

**Table 4:** Variables predicting PF score: results of multiple linear regression models.

Categories	All		Boys		Girls	
	B	OR (95% CI)	B	OR (95% CI)	B	OR (95% CI)
**Gender**						
Boys	0 ^a^	1.0 (Referent)				
Girls	3.27	26.38 (16.97, 41.01) ***				
**Age**	1.46	4.29 (3.80, 4.85) ***	1.88	6.56 (5.41, 7.94) ***	1.07	2.92 (2.50, 3.40) ***
**Place of Residence**						
Urban	0 ^a^	1.0 (Referent)	0 ^a^	1.0 (Referent)	0 ^a^	1.0 (Referent)
Rural	1.44	4.24 (2.66, 6.75) ***	2.08	8.00 (3.89, 16.45) ***	0.77	2.15 (1.19, 3.87) *
**Father’s educational level**						
Junior high school and below	0 ^a^	1.0 (Referent)	0 ^a^	1.0 (Referent)	0 ^a^	1.0 (Referent)
High school	−0.054	0.95 (0.50, 1.78)	0.039	1.04 (0.39, 2.76)	−0.31	0.74 (0.33, 1.65)
University and above	1.20	3.30 (1.51, 7.23) **	1.47	4.36 (1.32, 14.36) *	0.88	2.42 (0.89, 6.61)
**Mother’s educational level**						
Junior high school and below	0 ^a^	1.0 (Referent)	0 ^a^	1.0 (Referent)	0 ^a^	1.0 (Referent)
High school	−0.64	0.53 (0.27, 1.02)	−0.81	0.45 (0.16, 1.25)	−0.38	0.69 (0.30, 1.59)
University and above	−0.25	0.78 (0.35, 1.77)	−1.52	0.22 (0.063, 0.76) *	0.94	2.57 (0.90, 7.31)
**Family economic status**						
Poor	0 ^a^	1.0 (Referent)	0 ^a^	1.0 (Referent)	0 ^a^	1.0 (Referent)
Middle	0.33	1.39 (0.76, 2.55)	0.28	1.32 (0.53, 3.30)	0.37	1.45 (0.66, 3.16)
Good	0.41	1.51 0.57, 3.98)	1.25	3.48 (0.82, 14.74)	−0.66	0.52 (0.14, 1.87)
**PA**						
Inactive	0 ^a^	1.0 (Referent)	0 ^a^	1.0 (Referent)	0 ^a^	1.0 (Referent)
Active	0.99	2.69 (1.72, 4.20) ***	1.12	3.06 (1.53, 6.13) **	0.77	2.15 (1.23, 3.77) **
**Diet**						
GDR-Limit score	0.027	1.03 (0.99, 1.07)	0.023	1.02 (0.97, 1.09)	0.024	1.02 (0.97, 1.08)
GDR-Healthy score	0.023	1.02 (0.98, 1.07)	0.013	1.01 (0.95, 1.08)	0.028	1.03 (0.97, 1.09)

^a^ These parameters were set to 0 because they were redundant. Bonferroni *p*-values were used to correct for multiple comparisons. * *p* < 0.05, ** *p* < 0.01, *** *p* < 0.001.

**Table 5:** Linear regression analysis between dietary patterns and physical fitness.

PF Test	DP	All		Boys		Girls	
		Model 1	Model 2	Model 1	Model 2	Model 1	Model 2
**Body Mass Index**	DP1	1.06 (0.97, 1.15)	1.06 (0.97, 1.15)	1.01 (0.89, 1.14)	1.01 (0.89, 1.14)	1.12 (1.00, 1.26)	1.12 (1.001, 1.26) *
	DP2	0.84 (0.74, 0.94) **	0.84 (0.75, 0.95) **	0.84 (0.70, 1.01)	0.85 (0.71, 1.02)	0.84 (0.72, 0.97) *	0.84 (0.72, 0.98) *
	DP3	1.06 (0.96, 1.18)	1.08 (0.97, 1.19)	1.13 (0.97, 1.32)	1.15 (0.98, 1.34)	0.99 (0.86, 1.14)	1.00 (0.87, 1.16)
	DP4	1.09 (0.96, 1.23)	1.08 (0.96, 1.22)	1.06 (0.88, 1.28)	1.05 (0.87, 1.27)	1.12 (0.96, 1.30)	1.11 (0.96, 1.30)
**Vital Capacity score**	DP1	0.90 (0.60, 1.35)	0.90 (0.60, 1.35)	0.56 (0.31, 0.98) *	0.56 (0.32, 0.98) *	1.51 (0.84, 2.71)	1.50 (0.84, 2.68)
	DP2	1.09 (0.62, 1.92)	1.10 (0.62, 1.94)	1.01 (0.44, 2.31)	1.05 (0.46, 2.41)	1.14 (0.53, 2.47)	1.12 (0.52, 2.43)
	DP3	0.89 (0.54, 1.47)	0.91 (0.55, 1.50)	1.37 (0.68, 2.76)	1.48 (0.73, 2.99)	0.57 (0.28, 1.16)	0.56 (0.28, 1.14)
	DP4	0.82 (0.46, 1.45)	0.81 (0.46, 1.44)	1.03 (0.44, 2.40)	1.00 (0.43, 2.32)	0.67 (0.31, 1.46)	0.67 (0.31, 1.47)
**50 m Sprint score**	DP1	1.03 (0.67, 1.57)	1.02 (0.67, 1.56)	0.75 (0.41, 1.36)	0.75 (0.41, 1.36)	1.51 (0.83, 2.76)	1.49 (0.82, 2.73)
	DP2	1.40 (0.77, 2.53)	1.36 (0.75, 2.46)	1.64 (0.68, 3.94)	1.59 (0.66, 3.82)	1.19 (0.54, 2.65)	1.17 (0.52, 2.60)
	DP3	2.03 (1.20, 3.42) **	1.94 (1.15, 3.28) *	1.81 (0.86, 3.79)	1.70 (0.81, 3.58)	2.37 (1.14, 4.95) *	2.31 (1.11, 4.84) *
	DP4	0.96 (0.53, 1.75)	0.97 (0.54, 1.77)	0.75 (0.31, 1.82)	0.77 (0.31, 1.87)	1.23 (0.55, 2.76)	1.23 (0.55, 2.76)
**Sit and Reach score**	DP1	1.14 (0.75, 1.73)	1.13 (0.74, 1.71)	1.27 (0.73, 2.20)	1.26 (0.73, 2.19)	0.94 (0.50, 1.78)	0.93 (0.49, 1.75)
	DP2	2.36 (1.32, 4.23) **	2.28 (1.28, 4.08) **	3.03 (1.36, 6.77) **	2.90 (1.30, 6.49) **	2.00 (0.87, 4.63)	1.94 (0.84, 4.50)
	DP3	0.64 (0.39, 1.07)	0.61 (0.36, 1.02)	0.79 (0.40, 1.55)	0.73 (0.37, 1.44)	0.52 (0.24, 1.13)	0.50 (0.23, 1.09)
	DP4	0.60 (0.33, 1.08)	0.61 (0.34, 1.09)	0.34 (0.15, 0.76) **	0.35 (0.15, 0.79) *	1.04 (0.45, 2.42)	1.04 (0.45, 2.42)
**Standing Long Jump score**	DP1	0.78 (0.49, 1.23)	0.77 (0.48, 1.21)	0.53 (0.27, 1.02)	0.53 (0.27, 1.01)	1.19 (0.63, 2.24)	1.13 (0.60, 2.14)
	DP2	1.65 (0.87, 3.12)	1.54 (0.81, 2.91)	1.85 (0.71, 4.82)	1.74 (0.67, 4.55)	1.41 (0.61, 3.28)	1.30 (0.56, 3.02)
	DP3	1.37 (0.78, 2.40)	1.23 (0.70, 2.16)	1.05 (0.47, 2.36)	0.94 (0.42, 2.13)	1.99 (0.92, 4.32)	1.79 (0.83, 3.91)
	DP4	0.62 (0.33, 1.18)	0.64 (0.34, 1.22)	0.82 (0.31, 2.18)	0.86 (0.33, 2.28)	0.49 (0.21, 1.14)	0.49 (0.21, 1.15)
**1000 m Run score**	DP1			1.36 (0.64, 2.91)	1.36 (0.64, 2.89)		
	DP2			1.88 (0.62, 5.70)	1.66 (0.55, 5.04)		
	DP3			0.87 (0.34, 2.22)	0.69 (0.27, 1.79)		
	DP4			0.54(0.18, 1.67)	0.60 (0.19, 1.84)		
**Pull-ups score**	DP1			0.92 (0.79, 1.07)	0.92 (0.79, 1.07)		
	DP2			0.91 (0.78, 1.07)	0.92 (0.78, 1.08)		
	DP3			1.15 (1.04, 1.27) **	1.18 (1.06, 1.30) **		
	DP4			1.05 (0.96, 1.14)	1.04 (0.96, 1.14)		
**800 m Run** **score**	DP1					0.67 (0.33, 1.36)	0.65 (0.32, 1.32)
	DP2					1.66 (0.65, 4.27)	1.57 (0.61, 4.04)
	DP3					1.35 (0.57, 3.20)	1.25 (0.53, 2.99)
	DP4					0.87 (0.34, 2.25)	0.87 (0.34, 2.26)
**1-Minute Sit-Up** **score**	DP1					1.27 (0.69, 2.34)	1.27 (0.69, 2.34)
	DP2					1.14 (0.51, 2.56)	1.14 (0.51, 2.57)
	DP3					1.27 (0.61, 2.67)	1.28 (0.61, 2.69)
	DP4					0.60 (0.27, 1.35)	0.60 (0.26, 1.35)

* *p* < 0.05, ** *p* < 0.01. Model 1: Adjusted by age, place of residence, parents’ educational level and family economic status. Model 2: Additionally adjusted by physical activity.

## Data Availability

The data presented in this study are openly available in the Population Health Data Archive [34].
